# Supplementary material for: Impact of COVID-19 Diagnosis on Mortality in Patients with ST-Elevation Myocardial Infarction Hospitalized during the National Outbreak in Italy
Source: J Clin Med. 2022 Dec 10;11(24):7350. doi: 10.3390/jcm11247350 (PMC9787833; doi:10.3390/jcm11247350)
Supplement: Supplementary file 1 [file jcm-11-07350-s001.zip › jcm-2009926-Supplementary.pdf]

Supplementary Table S1. ICD9-CM codes used to retrieve information on risk factors and comorbidities.

	ICD9 Code	
	**Index admission**	**Previous admission**
Hypertension		401-405
Diabetes		250.0-250.9
Heart failure		428
Ill-defined descriptions and complications of heart disease		429
Cerbrovascular disease	433, 437, 438	430-432, 433, 434, 436, 437, 438
Vascular disease	440-448 (except 441.1, 441.3, 441.5, 441.6, 444), 557.1	440-448, 557
Chronic coronary syndromes		411, 413, 414
Arrhythmias		426, 427
Anemia	280-284, 285 (except 285.1)	280-284, 285 (except 285.1)
Blood clotting defects	286	286
Other hematological diseases	287-289	287-289
Cardiomyopathy	425	425
Rheumatic heart disease	393-398	391, 393-398
Endocarditis and acute myocarditis		421, 422
Other chronic heart conditions	745, V15.1, V42.2, V43.2, V43.3, V45.0	745, V15.1, V42.2, V43.2, V43.3, V45.0
Chronic kidney diseases	582, 583, 585-588	582, 583, 585-588
Chronic disease (liver, pancreas, intestine)	571, 572, 577.1-577.9, 555, 556	571, 572, 577.1-577.9, 555, 556
Obesity	278,0	278,0
Chronic obstructive pulmonary disease		491, 492, 494, 496
Malignant neoplasms	140.0–208.9, V10	140.0–208.9, V10
Previous vascular surgery		38-39.5 (except: 38.01, 38.02, 38.5, 38.11, 38.12, 38.31, 38.32)
Previous cerebral revascularization		00.61, 00.62, 38.01, 38.02, 38.11, 38.12, 38.31, 38.32
Other previous cardiac surgery than CABG		35, 37.0, 37.1, 37.3, 37.4, 37.5, 37.6, 37.9

Supplementary Table S2 Baseline characteristics of STEMI patients enrolled in 2020 with and without a COVID-19 diagnosis.

	**NO COVID (N=3878)**	**COVID (N=170)**	**P value**
Age (years), mean±SD	66.6±13.0	70.5±11.7	<0.0001
Gender (female), n (%)	979 (25.2)	49 (28.8)	0.29
Hypertension, n (%)	447 (11.5)	28 (16.5)	0.05
Diabetes, n (%)	250 (6.4)	15 (8.8)	0.22
Heart failure, n (%)	136 (3.5)	8 (4.7)	0.41
Ill-defined descriptions and complications of heart disease, n (%)	29 (0.7)	1 (0.6)	0.81
Cerebrovascular disease, n (%)	159 (4.1)	14 (8.2)	0.009
Cerebrovascular disease (ind. adm.), n (%)	85 (2.2)	1 (0.6)	0.16
Vascular disease, n (%)	127 (3.3)	7 (4.1)	0.55
Vascular disease (ind. adm.), n (%)	77 (2.0)	2 (1.2)	0.45
Chronic coronary syndromes, n (%)	303 (7.8)	12 (7.1)	0.72
Arrhythmias, n (%)	185 (4.8)	11 (6.5)	0.31
Anemia, n (%)	105 (2.7)	12 (7.1)	0.001
Anemia (ind. adm.) , n (%)	88 (2.3)	5 (2.9)	0.57
Blood clotting defects, n (%)	2 (0.1)	0 (0.0)	0.77
Blood clotting defects (ind. adm.), n (%)	1 (0.0)	0 (0.0)	0.83
Other hematological diseases, n (%)	17 (0.4)	0 (0.0)	0.39
Other hematological diseases (ind. adm.), n (%)	22 (0.6)	0 (0.0)	0.32
Cardiomyopathy, n (%)	15 (0.4)	0 (0.0)	0.42
Cardiomyopathy (ind. adm.), n (%)	59 (1.5)	1 (0.6)	0.32
Rheumatic heart disease, n (%)	14 (0.4)	0 (0.0)	0.43
Rheumatic heart disease (ind. adm.), n (%)	21 (0.5)	1 (0.6)	0.93
Endocarditis and acute myocarditis, n (%)	3 (0.1)	0 (0.0)	0.72
Other chronic heart conditions, n (%)	28 (0.7)	0 (0.0)	0.27
Other chronic heart conditions (ind.adm.), n (%)	42 (1.1)	1 (0.6)	0.54
Chronic kidney disease, n (%)	129 (3.3)	10 (5.9)	0.07
Chronic kidney diseases (ind. adm.), n (%)	242 (6.2)	6 (3.5)	0.15

Other chronic disease (liver, pancreas, intestine), n (%)	38 (1.0)	2 (1.2)	0.80
Other chronic disease (liver, pancreas, intestine) (ind. adm.), n (%)	10 (0.3)	1 (0.6)	0.42
Obesity, n (%)	45 (1.2)	4 (2.4)	0.16
Obesity (ind. adm.), n (%)	98 (2.5)	2 (1.2)	0.27
Chronic obstructive pulmonary disease, n (%)	99 (2.6)	5 (2.9)	0.75
Malignant neoplasms, n (%)	257 (6.6)	16 (9.4)	0.16
Previous MI	321 (8.3)	20 (11.8)	0.11
Previous vascular surgery, n (%)	131 (3.4)	7 (4.1)	0.60
Previous cerebral revascularization, n (%)	17 (0.4)	1 (0.6)	0.77
Previous CABG	76 (2.0)	6 (3.5)	0.15
Other previous cardiac surgery than CABG, n (%)	43 (1.1)	0 (0.0)	0.17
Previous PCI	486 (12.5)	24 (14.1)	0.54

CABG: coronary artery bypass grafting; MI: myocardial infarction; PCI: percutaneous coronary intervention;
Note: ind. adm - comorbidity information retrieved at the index admission.

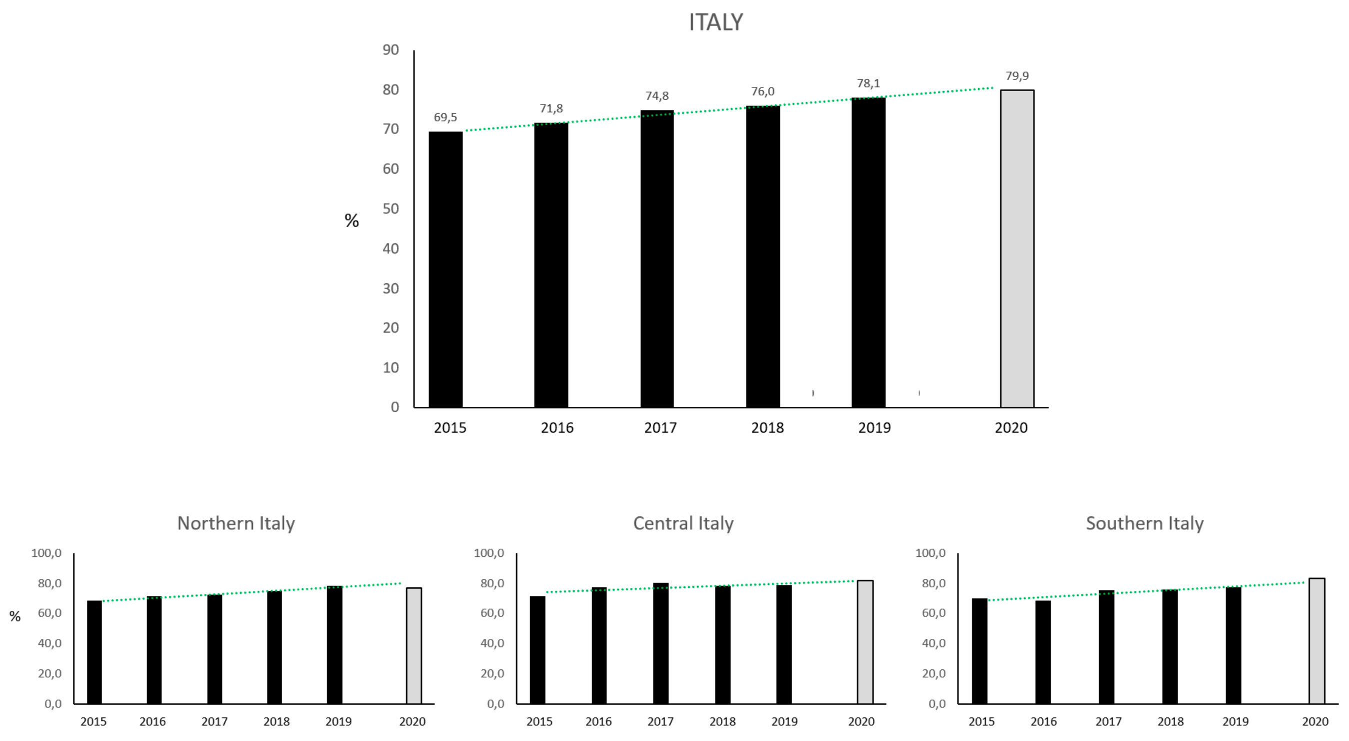

Supplementary Figure S3. Rates of PCI performed ≤2 days from hospital admission during the 2020 national outbreak and over the equivalent periods in the previous 5 years in Italy and by geographic regions.

Supplementary Table S3. Logistic regression model for 30 days mortality.

	**Crude OR**	**Adjusted OR**	**CI 95%**	**P value**
Age (years)	1.1	1.1	1.066 - 1.074	<0.0001
Gender (females)	2.6	1.2	1.126 - 1.329	<0.0001
Covid-19	4.3	4.5	3.098 - 6.450	<0.0001
Chronic coronary syndromes	2.0	1.3	1.151 - 1.516	<0.0001
Other previous cardiac surgery than CABG	1.8	1.6	1.140 - 2.195	0.006
Previous vascular surgery	2.5	1.5	1.228 - 1.721	<0.0001
Anemia (ind. adm.)	1.4	0.6	0.445 - 0.686	<0.0001
Obesity (ind. adm.)	0.4	0.6	0.447 - 0.901	0.011
Diabetes	2.3	1.2	1.053 - 1.357	0.006
Heart failure	3.9	1.3	1.156 - 1.557	<0.0001
Cerebrovascular disease	3.0	1.2	1.091 - 1.413	<0.0001
Chronic kidney disease	3.5	1.3	1.091 - 1.512	0.003
Previous PCI	0.5	0.4	0.386 - 0.518	<0.0001
Malignant neoplasms	2.1	1.4	1.222 - 1.561	<0.0001
Previous CABG	0.7	0.5	0.414 - 0.719	<0.0001
PCI ≤2 days	0.2	0.5	0.450 - 0.530	<0.0001

CABG: coronary artery bypass grafting; PCI: percutaneous coronary intervention;
Note: ind. adm - comorbidity information retrieved at the index admission
